# Retinoic Acid Regulates Allergic Inflammation via Limiting Mast Cell Activation

**DOI:** 10.1002/fsn3.4727

**Published:** 2025-01-07

**Authors:** Wenxin Zhang, Wenwen Dai, Yingdong Xie, Xingyang Chen, Peng Zhang, Weiwei Cui

**Affiliations:** ^1^ Department of Pathology The First Hospital of Jilin University Changchun China; ^2^ Department of Nutrition and Food Hygiene, School of Public Health Jilin University Changchun China; ^3^ Department of Thoracic Surgery The First Hospital of Jilin University Changchun China

**Keywords:** allergic diseases, mast cell, RAR, retinoic acid receptor, vitamin A

## Abstract

BackgroundAllergic diseases have become one of the major public health problems to be addressed in the world today. As a tissue resident cell, mast cells are crucial in the pathogenesis of allergic diseases. Vitamin A is an important fat‐soluble vitamin with immunomodulatory functions. Vitamin A deficiency has been shown to be associated with allergic disease states, including asthma; however, no studies have been reported on whether vitamin A deficiency has an effect on the activation of mast cells in allergic reactions. ObjectiveTo explore whether blocking retinoic acid receptors has an effect on mast cell degranulation. Methods Flow cytometry was used to analyze the expression of FCεRIα and CD117 on the cell surface, toluidine blue staining was used to visualize cellular features and morphological changes. ELISA was used to detect histamine release. High‐throughput transcriptome sequencing and qRT‐PCR were used to detect the expression of relevant signaling pathways and cytokine genes. Western blot was used to detect the expression of relevant signaling pathway proteins. ResultsIn the present study, we found that antagonism of the retinoic acid receptor (RAR) resulted in overactive mast cells and increased their degranulation. Furthermore, inflammatory signaling pathways such as MyD88‐IKK‐NF‐κB and PI3K‐Akt‐m‐TOR were involved in the effect of retinoic acid (RA) on the activation state of mast cells. ConclusionsIn this paper, we demonstrated that blocking RAR can exacerbate its activation state in IgE‐mediated mast cells. This study provided new insights into the possibility that vitamin A deficiency exacerbated mast cell activation and thus affectd allergic diseases and their mechanisms.

## Graphical abstract

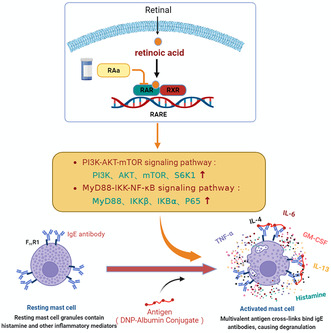

## 1 Introduction

According to a World Allergy Organization (WAO) survey of 30 countries/regions, approximately 250 million of the population suffers from allergic diseases that include asthma, food allergies, hives, and drug allergies, et al. (Pawankar et al. 2013). While a large body of evidence suggests that Immunoglobulin E (IgE) and mast cells are key drivers in allergic disease, mast cells playing an important role in the induction of allergic inflammation by releasing various mediators, including lipid mediators, chemokines and cytokines, as important effector cells (Galli and Tsai 2012; Gilfillan and Beaven 2011). Mast cell activation can also occur secondary to allergic, inflammatory, or paraneoplastic disease, with consequences including urticaria, allergic rhinitis, and asthma (Jackson et al. 2021; Akin 2017). In addition to their prominent role in allergic responses, mast cells also have complex interactions with innate immunity, adaptive immunity, immune tolerance, angiogenesis, tumor surveillance, in vivo homeostasis, and tissue repair in defense against pathogens, wound healing, and tumor surveillance (Da Silva, Jamur, and Oliver 2014; Gri et al. 2012; Varricchi et al. 2019; Henz et al. 2001).

Vitamin A (all‐trans retinol) is a fat‐soluble micronutrient that constitutes the retinol group together with its derivatives (McLaren and Kraemer 2012). The retinaldehyde (RAL) and retinoic acid (RA) that are converted from vitamin A in the form of all‐trans or isomers through oxidation reaction become the main biologically active derivatives and perform various important physiological functions of vitamin A in the human body (Timoneda et al. 2018). In the eye, it is mainly RAL that binds to optoprotein and thus maintains vision; in most cells, the main metabolite of vitamin A is retinoic acid, which binds to two types of transcription factors (RARs and retinoid X receptors) and exerts most of the biological effects of vitamin A (Cabezuelo et al. 2019). Vitamin A can alleviate the occurrence of allergic rhinitis and asthma by reducing the degree of inflammatory response (Feng et al. 2021). Marquez et al. found that the anomalies of differentiation, development and function of pulmonary airway smooth muscle due to the deficiency of vitamin A is associated with allergic disease states including asthma (Marquez and Cardoso 2016). Our previous results also indicated that vitamin A deficiency (VAD) exacerbated ovalbumin‐induced asthma and lung inflammation by enhancing inflammatory cell infiltration and significantly increased levels of type 2 cytokines, particularly interleukin‐5 (IL‐5) and IL‐13 (Cui et al. 2016). At present, it has been shown that a mouse model of VAD shown significant mast cell aggregation and elevated IgE in serum (Yang et al. 2020). In addition, studies from animal models have shown that vitamin A maintains mast cell stability and thus alleviates the release of allergic mediators (Hufnagl and Jensen‐Jarolim 2018).

Although several studies have suggested a possible association between vitamin A, mast cells, and allergic diseases, it is not known whether vitamin A deficiency can affect allergic diseases by mediating mast cell function and the mechanisms involved. Based on the above, we hypothesized that vitamin A deficiency could lead to IgE‐mediated mast cell activation. To test this hypothesis, we investigated whether VAD exacerbates IgE‐mediated mast cell activation and explored its mechanism by designing corresponding experimental studies to antagonize RAR, thus mimicking the state of VAD to a certain extent. The study of intrinsic mechanisms is used to investigate the association between VAD and allergic diseases, thus providing a theoretical basis for the study of allergic diseases.

## 2 Results

### 2.1 Identification of Mouse Bone Marrow‐Derived Mast Cells

**FIGURE 1:**
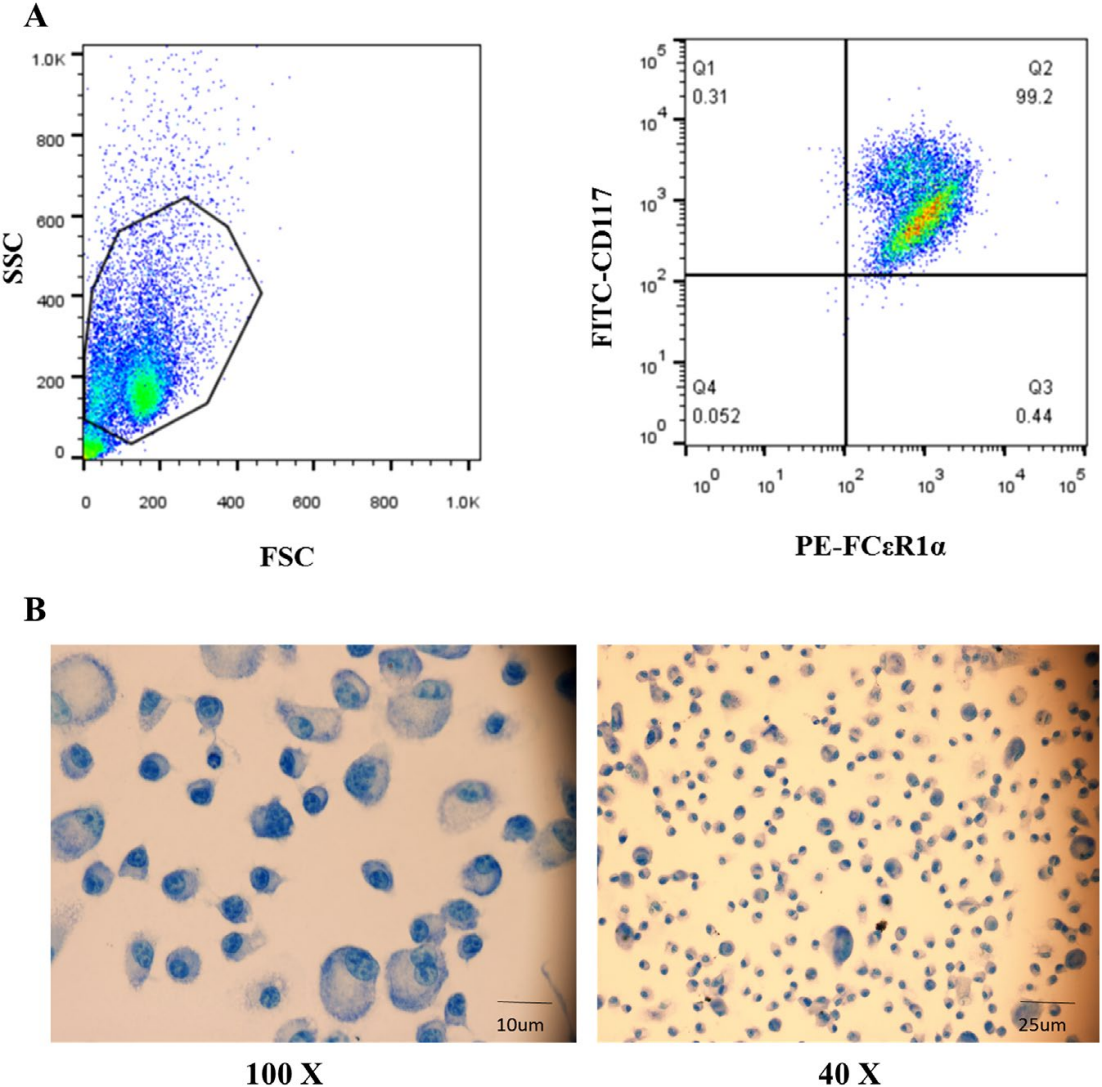
Identification of mouse bone marrow‐derived mast cells. (A) Bone marrow‐derived mast cells identification by flow cytometry. (B) Toluidine blue staining for identification of bone marrow‐derived mast cells.

To verify whether the cells that were obtained from bone marrow hematopoietic stem cells of C57BL/6J mice and induced by IL‐3 for 4 weeks were mast cells, flow cytometry and toluidine blue staining were performed. As shown in Figure 1A, CD117 and FcεR1α double‐positive cells with a purity of 99.2% could be obtained by flow cytometry. In addition, we stained the primary cells after 4 weeks of culture with toluidine blue, and the results showed that the nucleus was stained blue and the cytoplasm was stained purple, which was consistent with the characteristics of mast cells (Figure 1B). Based on the above results, follow‐up experiments were performed with BMMCs from C57BL/6J mice.

### 2.2 Antagonizing the RAR Aggravated IgE‐Mediated BMMC Degranulation

**FIGURE 2:**
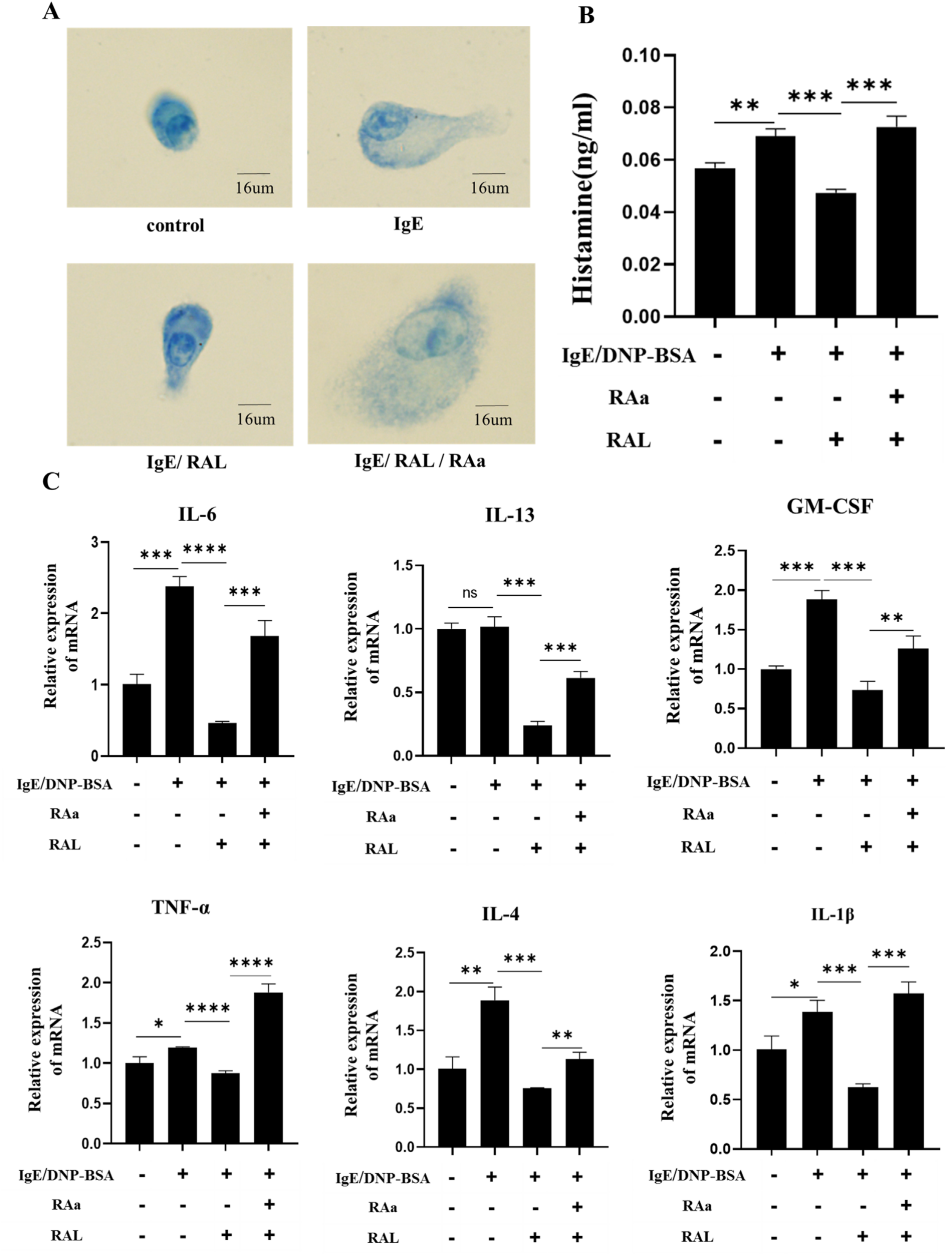
Effects of vitamin A intervention on the morphology and degranulation of BMMCs. (A) Morphological changes of BMMCs after RAL intervention were determined by toluidine blue staining. (B) The concentration of histamine released from BMMCs was detected by ELISA. (C) The gene expression levels of IL‐6, IL‐13, GM‐CSF, TNF‐α, IL‐4 and IL‐1β in BMMCs after RAL intervention were determined by qRT‐PCR. Data represented as means ± SD (*n* > 6), Significant differences between the groups statistically analyzed using analysis of variance (ANOVA) and LSD methods, *indicates *p* < 0.05, **indicates *p* < 0.01, ***indicates *p* < 0.001, ****indicates *p* < 0.0001 and ns indicates no significance.

We next examined the antiallergic effect of vitamin A on IgE‐induced mast cell degranulation by toluidine blue staining, enzyme‐linked immunosorbent assay (ELISA), and quantitative real‐time polymerase chain reaction (qRT‐PCR). As shown in Figure 2A, typical morphologic changes in mast cells, such as increased of cell volume and degranulation, were observed in IgE group compared with Control group. After RAL intervention, the cell volume and degree of degranulation in the IR group were generally lower than those in the IgE group, while the IRR group counteracted some of the effects of RAL compared to the IR group. By detecting the alteration of histamine concentration and cytokine expression in the control and experimental groups, we found that ELISA results showed an increased level of histamine secretion in the IgE group compared to the control group and qRT‐PCR assays showed that the increased secretion levels of particle markers such as histamine, IL‐6, IL‐13, granulocyte macrophage‐colony stimulating factor (GM‐CSF), tumor necrosis factor‐α (TNF‐α), IL‐4, and IL‐1β in the IgE group compared with Control group. The results that histamine, IL‐6, IL‐13, GM‐CSF, TNF‐α, IL‐4, and IL‐1β decreased by 31.33%, 80.55%, 76.44%, 60.84%, 26.68%, 60.00%, and 54.75% (*p* < 0.05) were observed respectively in IR group after RAL intervention, while the phenomenon was counteracted in the IRR group after RAL action was blocked (Figure 2B,C). All of the above results can demonstrate that blocking the RAR aggravated IgE‐mediated BMMC degranulation.

### 2.3 Effect of Antagonizing the RAR on NF‐κB and m‐TOR Signaling Pathway by RNA Sequencing

**FIGURE 3:**
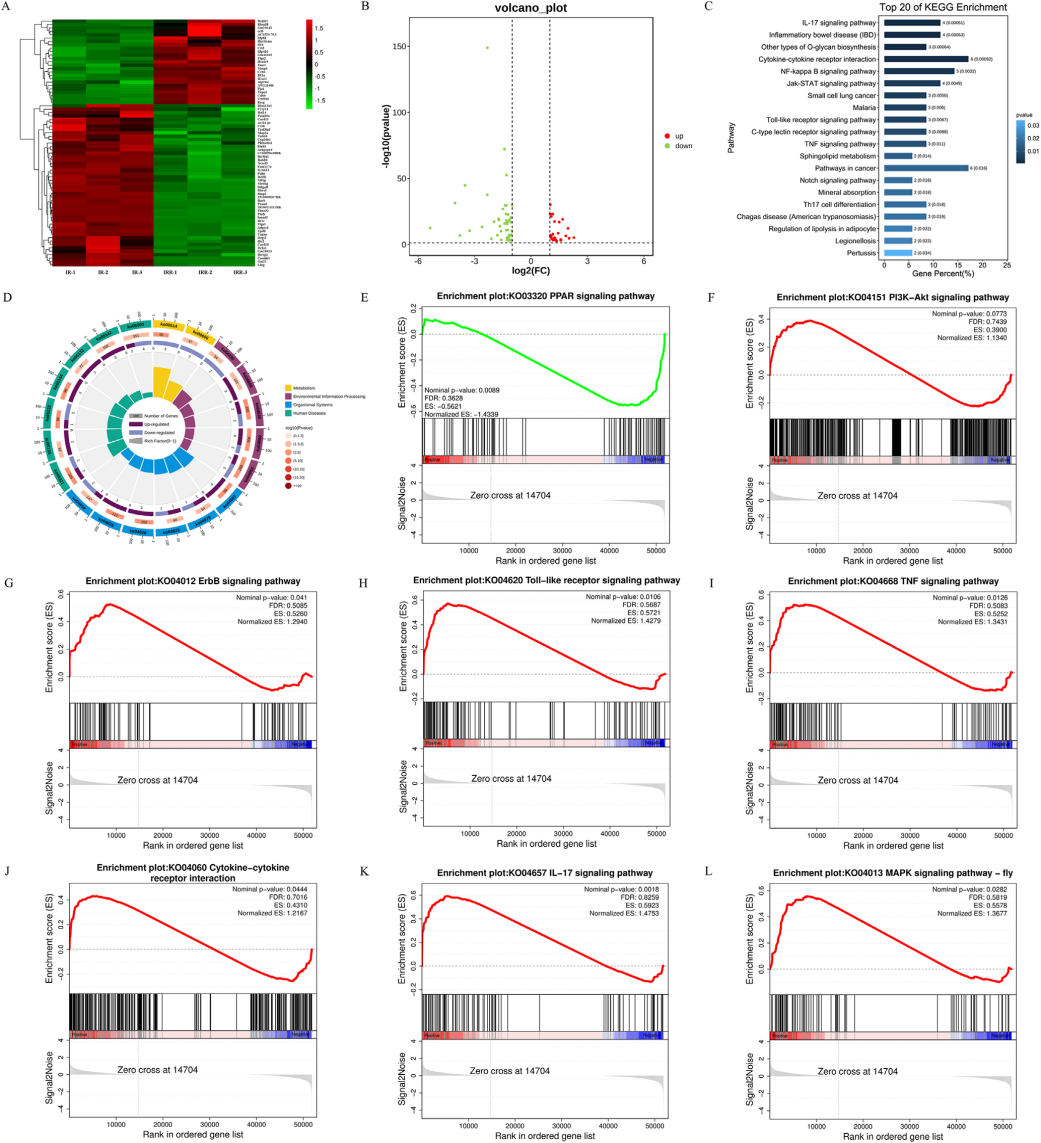
High‐throughput gene sequencing analysis of BMMC transcriptome. (A, B) Correlation heat map and volcano plot of gene expression differences after RAL intervention, left (i.e., IR group), right (i.e., IRR group). The experiments were repeated three times independently, and the up‐regulated genes and down‐regulated genes were highlighted in red and green. (C) KEGG enrichment analysis shown the top 20 pathway bar graphs of gene expression differences after RAL intervention. (D) Enrichment circle plot shown gene expression differences after RAL intervention. (E‐L) GSEA diagrams shown differences in gene expression of the PPAR, PI3K‐AKT, ErbB, Toll‐like receptor, TNF, Cytokine‐cytokine receptor interaction, IL‐17, MAPK signaling pathway. The experiments were repeated three times independently, and the up‐regulated genes and down‐regulated genes were highlighted in red and blue.

The differences of gene expression between IR and IRR groups were detected by high‐throughput transcriptome gene sequencing. As shown in Figure 3A,B, heatmap and volcano plot showed that a total of 73 differential genes were significantly expressed in the IRR group compared with the IR group, of which 25 genes were up‐regulated and 48 genes were down‐regulated. Since RA is thought to act through the RAR (Lee et al. 2017), we included retinoic acid receptor antagonist (RAa) to confirm whether these effects were induced through the RAR. The results showed that RARβ gene expression was significantly down‐regulated in the IRR group, indicating that the RAa employed in this study exerted an effect and that RAa blocked the effect of RA on mast cell activation (Figure 3A). The nuclear factor‐κB (NF‐κB) signaling pathway that were enriched with five of the differential genes between IR and IRR groups (*p* < 0.01) was found by bar graph of KEGG enrichment pathway (Figure 3C). Enrichment of differential genes in the four enrichment categories and change of up‐regulation and down‐regulation was showed in enrichment circle plot (Figure 3D), which will help us to see more clearly the effect of antagonizing the RAR on human functions and even human diseases. In addition, gene set enrichment analysis (GSEA) was performed on the total gene data of the IR group and IRR group in order to explore whether the effect of blocking the RAR on genes was activation or inhibition. The results showed that the PPAR signaling pathway (vitamin A metabolism‐related pathway) was inhibited (Figure 3E), proving that RAa played their role again. Furthermore, the pathways that are related to the pathogenesis of inflammation, such as the phosphatidylinositol 3‐kinase (PI3K)/protein kinase B (AKT), Toll‐like receptor, tumor necrosis factor (TNF), IL‐17, mitogen‐activated protein kinase (MAPK), and the epidermal growth factor receptor (ErbB) signaling pathway as well as cytokine‐cytokine receptor interaction, were all enhanced (*p* < 0.05) (Figure 3F–L). Among them, PI3K/AKT and MAPK can be used as the upstream of mammalian target of rapamycin (m‐TOR) signaling pathway. Surprisingly, no statistically significant results was shown in the NF‐κB signaling pathway (Figure S1I), but the overall trend was as expected and several of its upstream pathways were activated.

### 2.4 Antagonizing the RAR Up‐regulated the Gene Expression of NF‐κB and m‐TOR Signaling Pathways in BMMCs

**FIGURE 4:**
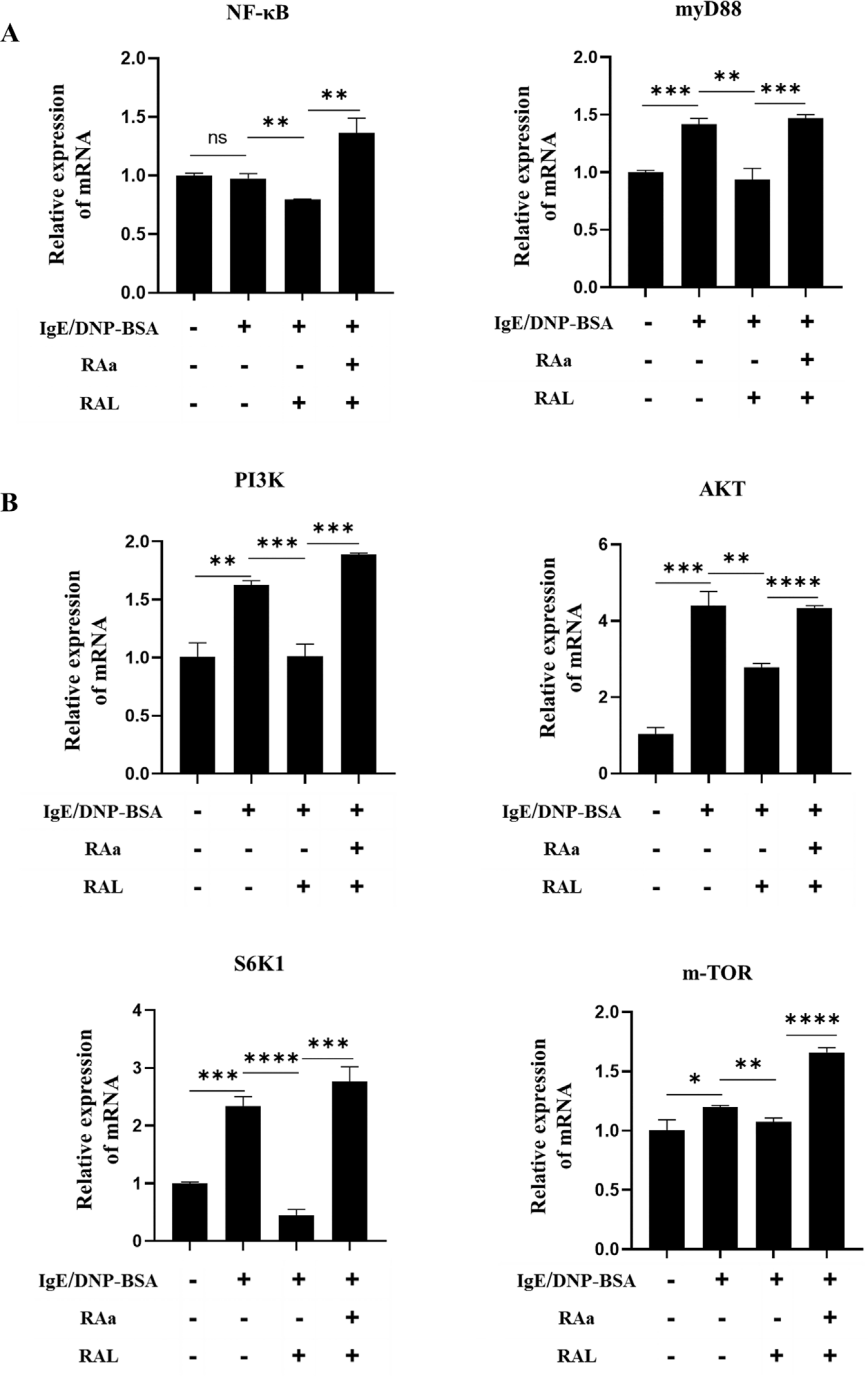
Expression of genes related to MyD88 and m‐TOR signaling pathways. (A) Genes expression of MyD88 and NF‐κB on the NF‐κB signaling pathway after RAL intervention using qRT‐PCR. (B) Genes expression of PI3K, AKT, m‐TOR on the PI3K‐AKT signaling pathway and their downstream S6K1 after RAL using qRT‐PCR. The experiments were repeated three times independently and data represented as means ± SD (*n* > 6), Significant differences between the groups statistically analyzed using analysis of variance (ANOVA) and LSD methods, *indicates *p* < 0.05, **indicates *p* < 0.01, ***indicates *p* < 0.001, ****indicates *p* < 0.0001 and ns indicates no significance.

Next, the expression of NF‐κB and m‐TOR signaling pathway‐related genes in BMMCs, including NF‐κB (P65), MyD88, PI3K, AKT, m‐TOR, and p70 ribosomal protein kinase 1 (S6K1) were detected by qRT‐PCR to further verify the molecular mechanisms by which blocking the RAR promotes BMMC degranulation. As shown in Figure 4A–C, qRT‐PCR assays showed that increased secretion levels of related signaling pathway markers (NF‐κB, MyD88, PI3K, AKT, m‐TOR, S6K1) in the IgE group compared with Control group. The results that NF‐κB, MyD88, PI3K, AKT, m‐TOR, and S6K1 decreased by 18.26%, 33.74%, 37.56%, 36.87%, 10.60%, and 81.13% (*p* < 0.05) were observed respectively in IR group after RAL intervention, while the effect was partially counteracted in the IRR group after RAL action was blocked. In conclusion, antagonizing the RAR up‐regulated the genes expression of MyD88‐IKK‐NF‐κB and PI3K‐AKT‐m‐TOR signaling pathways in BMMCs.

### 2.5 Antagonizing the RAR Up‐regulated the Protein Expression of NF‐κB and m‐TOR Signaling Pathways in BMMCs

**FIGURE 5:**
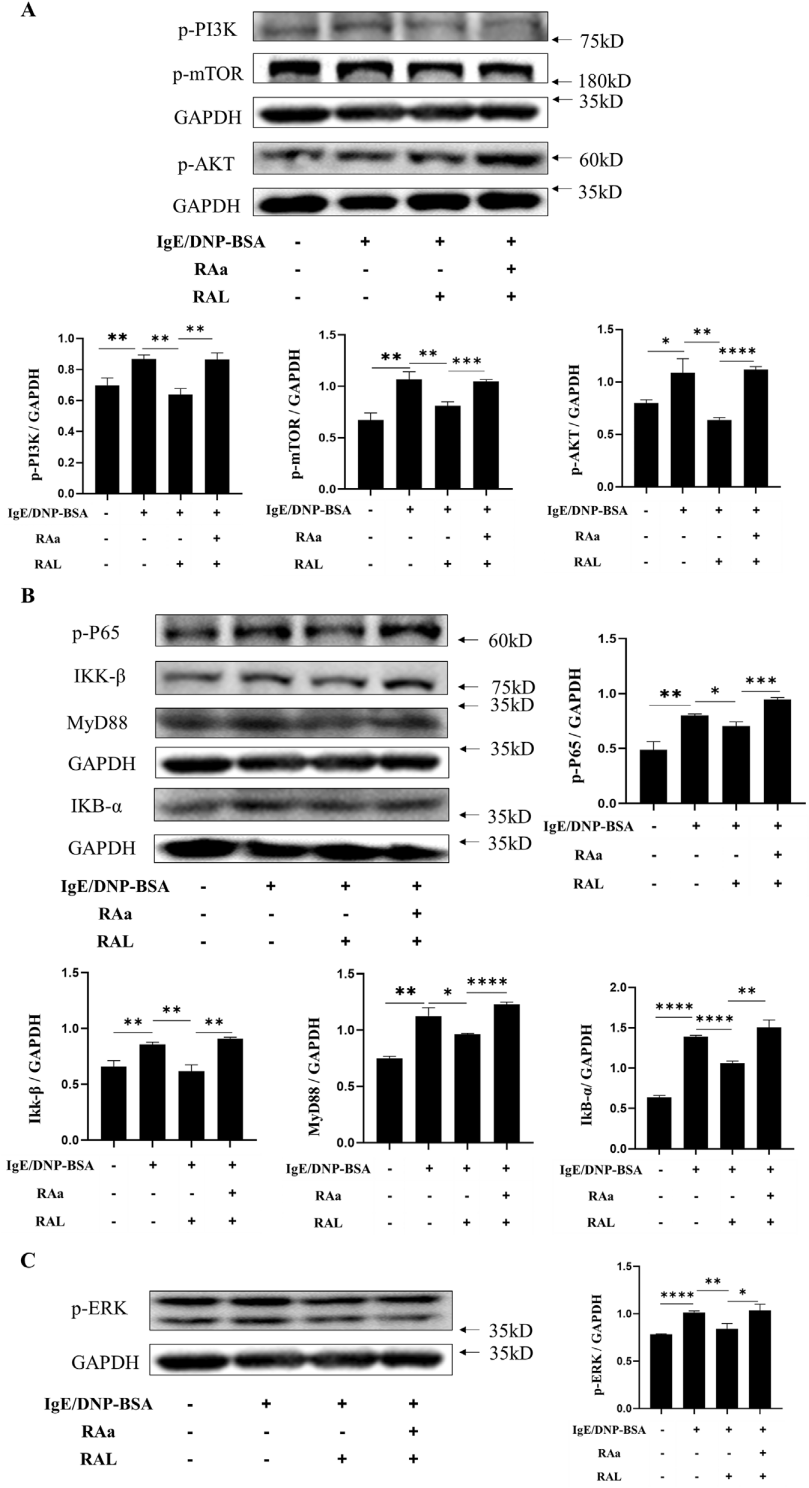
Effect of RAL on the expression of BMMC activation‐related proteins. BMMCs were treated with DMSO, anti‐DNP‐IgE (300 ng/mL), DNP‐BSA (300 ng/mL), RAa (3 μM), and RAL (0.3 μM) according to different groupings. Then, the cells were collected and proteins were extracted. The separate proteins were detected with enhanced chemiluminescence reagents. (A) Western blot analysis and quantitative analysis of PI3K‐AKT‐m‐TOR signaling pathway‐related protein expression. GAPDH was recruited as a loading control. (B) Western blot analysis and quantitative analysis of the expression of MyD88‐IKK‐NF‐κB signaling pathway‐related proteins. GAPDH was recruited as a loading control. (C) Western blot analysis and quantitative analysis of MAPK signaling pathway‐related protein expression. GAPDH was recruited as a loading control. The experiments were repeated three times independently and data were expressed as mean ± SD (*n* = 3). Significant differences between the groups statistically analyzed using analysis of variance (ANOVA) and LSD methods, *indicates *p* < 0.05, **indicates *p* < 0.01, ***indicates *p* < 0.001, ****indicates *p* < 0.0001 and ns indicates no significance.

In the above study, the genes expression of two signaling pathways were examined. Then, western blot was used to verify the inflammation‐related protein expression including p‐PI3K, p‐m‐TOR, p‐AKT, p‐P65, IKK‐β, MyD88, IKB‐α, and p‐ERK. As shown in the Figure 5A–C, the expression of p‐PI3K, p‐m‐TOR, p‐AKT, p‐P65, IKK‐β, MyD88, IKB‐α, and p‐ERK were significantly increased in BMMCs of IgE group compared with the control group (*p* < 0.05). The expression of these indicators was significantly lower in BMMCs in the IR group compared with the IgE group (*p* < 0.05). The phenomenon was counteracted in the IR group after the effect of RAR was blocked in the IRR group. These results are consistent with qRT‐PCR, suggesting that RAL alleviated IgE‐mediated activation of BMMCs and that RAa exacerbates the IgE‐induced activation state of BMMCs. Thus, it is suggested that antagonizing the RAR enhanced IgE‐mediated BMMCs activation by a mechanism involving the PI3K‐AKT‐m‐TOR and MyD88‐IKK‐NF‐κB pathways in inflammation.

## 3 Discussion

In this paper, the association between antagonizing the RAR and IgE‐mediated BMMCs activation were explored, and the data revealed a previously unrecognized effect and mechanism. Through our study, we found that mast cells pretreated with RAa showed significant increases in indicators of relevant inflammatory factors such as histamine, IL‐6, IL‐13, GM‐CSF, TNF‐α, IL‐4, and IL‐1β, thereby exacerbating the degranulation process of mast cells. We further found that these effects may be mediated through up‐regulation of NF‐κB/m‐TOR signaling pathway.

Vitamin A is an essential fat‐soluble micronutrient in our diet (Cabezuelo et al. 2019). It is widely believed that the most functions of vitamin A are performed by active oxidative metabolites produced in the target cells (Morita et al. 2019). VAD is one of the most common and important nutritional deficiency diseases worldwide, and research on VAD is of great importance for the governance of global public health (Long et al. 2021). As with the sequencing results, VAD was closely linked to signaling pathways related to inflammation and metabolism, and it may predispose the body to infectious and autoimmune diseases. Studies have shown that vitamin A can reduce the inflammatory response of allergic diseases and was an effective supplement for the treatment of asthma (Feng et al. 2021). Based on the long‐term in‐depth research on this topic, our previous study shown that VAD exacerbated ovalbumin‐induced asthma in mice, with increased lung inflammation and increased expression of type 2 cytokines and IgE, possibly due in part to group 2 innate lymphoid cells (ILC2s) proliferation and activation (Cui et al. 2016). Early asthma is highly IgE‐dependent and therefore IgE‐mediated mast cell degranulation is important for the development of asthma (Chen et al. 2021; Rakhmanova et al. 2020). The present study further found in vitro cytology that retinoic acid blockade led to the increase of volume and degranulation of mast cells, which in turn leads to mast cell hyper‐responsiveness, which is directly related to the development of allergic diseases.

In this paper, a cellular model of RAR blockade was established by using RAa that leads to a decrease in overall intracellular vitamin A levels. Mechanistically speaking, in most cells, the main physiological function performed is RA which in addition to binding to the so‐called RAR and can also activate another nuclear receptor, PPAR (Blatt et al. 2012). Although vitamin A may exert its physiological functions through a variety of pathways, in this experiment, we gave blockade to the main active substance retinoic acid, which can mimic VAD to some extent.

Cytokines secreted by mast cells play a very crucial role in the pathogenesis of allergic diseases and inflammation (Cantwell and Foreman 1989; Kastner et al. 2001). Our study showed that the addition of RAL resulted in a significant decrease of histamine, IL‐6, IL‐13, GM‐CSF, TNF‐α, IL‐4, and IL‐1β granules produced by mast cells, which was important for alleviating the sensitizing effect of the organism and mast cell activation. Histamine is the typical marker produced during the degranulation of mast cells (Iskakova et al. 2015; Sommer 1992). There is now evidence that IL‐6 can be produced by mast cell activation and degranulation, and it is also one of the key cytokines that promote mast cell maturation and histamine production in turn (Hatzivlassiou et al. 2010; Lertnimitphun et al. 2021). During allergic reactions, IL‐4 was rapidly produced within mast cells and rapidly released as mast cells degranulate to stimulate the body to produce an inflammatory response (Kastner et al. 2001; Berry and Noy 2009). IL‐4 has been shown to be the first cytokine produced by mast cells followed by its promotion of IL‐13 production, and together they play a prominent role in stimulating and maintaining allergic responses, among other things (Plaut et al. 1989; McLeod, Baker, and Ryan 2015). Mast cells are an important source of TNF‐α and IL‐1β that can be released immediately upon degranulation, and there was evidence that mast cell‐derived TNF‐α played a key role in models of asthma allergen response (Im et al. 2011; Żelechowska et al. 2021). The overexpression or release of these substances is an important cause of the onset and progression of allergic diseases. At present, some studies have used the changes of the immune system of asthma patients as new targets (such as IgE, IL‐4, IL‐5, etc.) to generate new biological agents, but the cost‐effectiveness still needs to be considered (Conti et al. 2002; Demopoulos, Antonopoulou, and Theoharides 2020).

Studies have found that the m‐TOR signaling pathway played an important role in the occurrence and development of asthma, and it has been confirmed that inhibiting the m‐TOR pathway has therapeutic significance for asthma (Wang, Saxon, and Diaz‐Sanchez 1999). There is increasing evidence that activation of m‐TORC1 (m‐TOR complex 1) was associated with downstream target ribosomal protein S6 and inflammatory responses (Toru et al. 1998). Mechanistically, the canonical TLR4‐MyD88‐MAPK and NF‐κB signaling pathways regulate their roles in the pathogenesis of inflammation as key upstream and downstream of the m‐TOR signaling pathway, respectively (Bischoff et al. 1999). Our results were closely related to the above indicators, which was confirmed by transcriptome gene sequencing, qRT‐PCR, and western blot results. NF‐κB is considered to be a central mediator of inflammatory processes and innate immunity, and activates survival genes and inflammatory promoters in specific ways (Oettgen 2011). NF‐κB signaling pathway can be activated through a variety of classical pathways, such as IL‐17 signaling pathway (Babu et al. 2011; Patel and Sur 2017), toll‐like receptor signaling pathway and TNF signaling pathway (Morjaria, Gnanakumaran, and Babu 2007) and so on. In our results, the above pathways were all up‐regulated. Although the up‐regulation of NF‐κB pathway was not obvious in sequencing, it was verified by qRT‐PCR and western blot.

In addition, we also found that a large number of Toll‐like receptors were highly activated when RAR was blocked. Previous studies have found that TLR4, as an important pattern recognition receptor, can trigger and exacerbate the progression of asthma, and the TLR4‐MyD88 pathway was involved in asthma‐related signal transduction (Ma et al. 2021; Newton et al. 2015). Multiple studies have shown that TLR4‐mediated signaling can activate NF‐κB in multiple ways (Zhou et al. 2018; Hoesel and Schmid 2013), and deletion of TLR4 or MyD88 attenuates airway inflammation and hyper‐responsiveness (Chen and Zhou 2015). Based on our results, we speculated that antagonizing the RAR can aggravate mast cell Activation, which may also be related to the over activation of TLRs and the exact mechanisms need to be studied in more depth. Our results also found that the PI3K‐AKT signaling pathway was up‐regulated in IRR group. and several studies have shown that both AKT and m‐TOR can promote the activation of IKK, which turns on NF‐κB (Reynolds, Angkasekwinai, and Dong 2010). In summary, we suggested that the molecular mechanism of promoting IgE‐mediated mast cell activation by antagonizing and RAR was similar to that of conventional allergy. Mast cell degranulation was progressively amplified by the interaction of multiple signaling pathways, the main enriched pathways being PI3K‐AKT‐m‐TOR and MyD88‐IKK‐NF‐κB, as verified in our results.

In conclusion, our findings provide new evidence that antagonizing RAR can lead to increased mast cell activation and that antagonizing the RAR can exacerbate the release of histamine and inflammatory cytokines from BMMC cells. Furthermore, in this paper, we found that MyD88‐IKK‐NF‐κB and PI3K‐AKT‐m‐TOR‐mediated inflammatory signaling pathways were involved in the effect of RA on BMMC activation.

**FIGURE 6:**
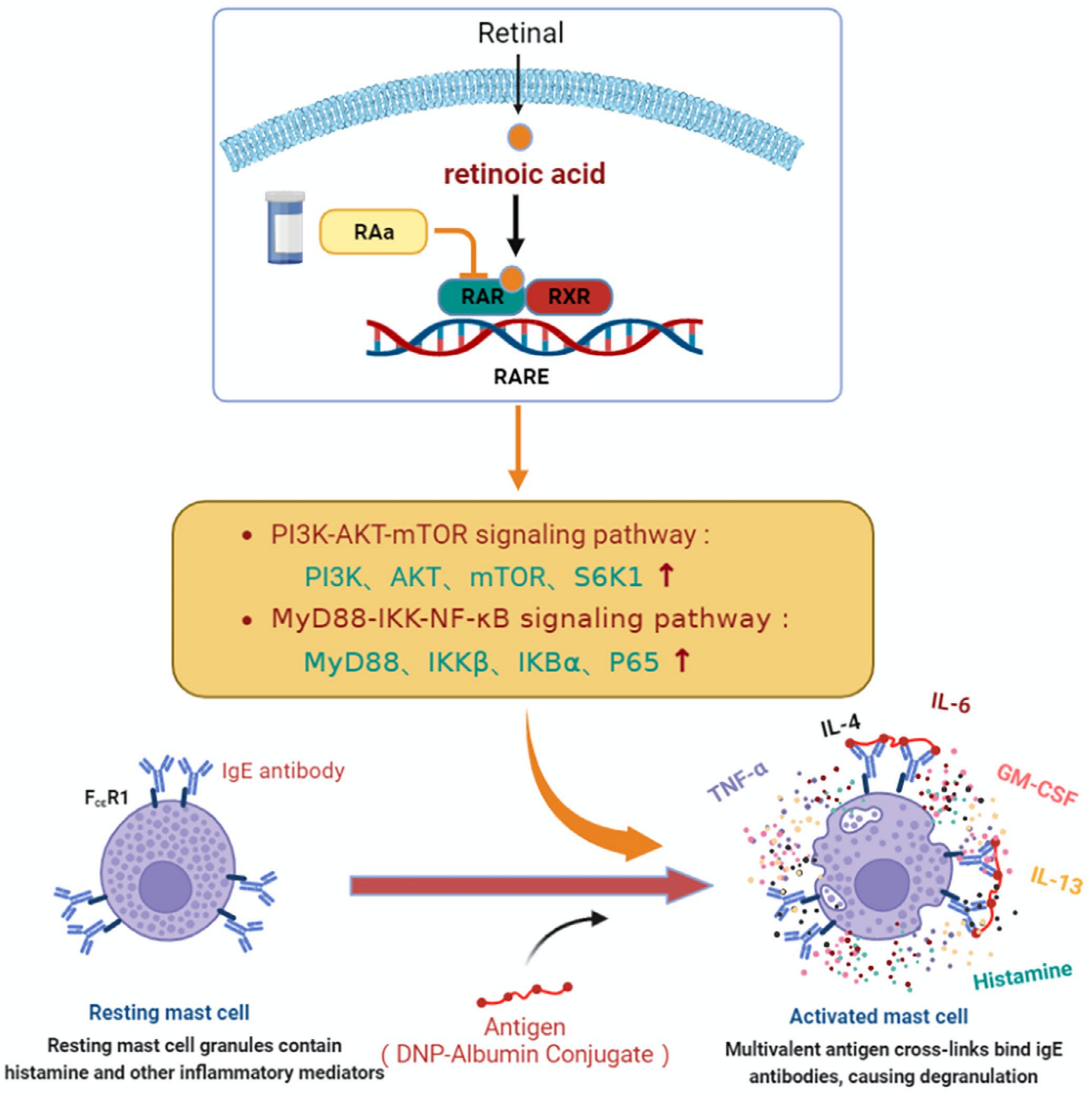
The mechanisms by which RAR receptor blockade exacerbated mast cell activation.

As summarized in Figure 6, we conducted this study by antagonizing RAR which to some extent mimics vitamin A deficiency, to explore the effect of vitamin A deficiency on mast cell activation, which not only provides a new research direction to study the clinical application of vitamin A, but also links vitamin A to allergic diseases, thus providing a new way to investigate the pathological process of allergic diseases and their treatment, ideas and data support. However, our experiments were based on cellular experiments and the scope of the study was relatively narrow, as the effect of RA that the active component of vitamin A, on the activation of mast cells was only discussed at the level of mast cells, thus it was a relatively basic experiment. Therefore, in order to investigate the association between VAD and allergic diseases, further experiments and more comprehensive experiments are needed in the future to demonstrate this, including animal experiments, randomized controlled experiments, and clinical trials.

## 4 Materials and Methods

### 4.1 Reagents and Antibodies

The adult C57BL/6J mice were ordered from Liaoning Changsheng Biotechnology Co. Ltd. (Nanjing, China). Fetal bovine serum (FBS) and RPMI‐1640 medium were purchased from Biological Industries (Kibbutz Beit Haemek, Israel). Recombinant Mouse IL‐3 was obtained from Absin (Shanghai, China). Toluidine blue staining solution (for cells) was got from Beijing Solarbio Science & Technology Co. Ltd. (Beijing, China). Phycoerythrobilin (PE)‐conjugated anti‐CD117 was procured from Bioss Biological Technology CO. Ltd. (Beijing, China) and FITC‐conjugated anti‐FCεR1α was procured from Invitrogen Corporation (Carlsbad, CA, USA) for use in flow cytometry. Mouse anti‐dinitrophenyl (DNP)‐IgE, DNP‐Bovine serum albumin (DNP‐BSA), all trans‐RAL, retinoic acid receptor antagonist (RAa, AGN194310, a pan‐RAR antagonist) were all purchased from Merck & Co Inc. (New Jersey, USA). ELISA kit for histamine was ordered from Elabscience (Wuhan, China).

### 4.2 Bone Marrow‐Derived Mast Cells (BMMCs) Culture

This experiment was reviewed by the Medical Ethics Committee of the School of Public Health, Jilin University, with the approval number (2017) Session Review No. (2017‐02‐09). After euthanasia of 8‐week‐old C57BL/6J mice, the skin of the abdomen and lower limbs was cut open, the leg muscles were separated from the skin, and the femur and tibia were cut from the ankle of the hind limb as well as from the root of the thigh. The muscles and ligaments were further stripped from the femur and tibia, washed twice in PBS and placed in a Petri dish containing RPMI‐1640 culture medium. The ends of the femur and tibia were cut and the bone marrow cells inside the bones were flushed with RPMI‐1640 culture medium using a syringe. After filtering and centrifuging the cells was cultured for at least 4 weeks in medium supplemented with 10% FBS, 90% RPMI‐1640, 1% penicillin–streptomycin mix (100×) at 37°C in 5% CO_2_. The medium was further supplemented with 10 ng/mL IL‐3 as a growth factor for murine mast cells (Schmid and Birbach 2008). The mast cell phenotype was confirmed by flow cytometry analysis of CD117 and FcεR1α‐specific antibodies and acidic toluidine blue histochemical staining.

### 4.3 Flow Cytometry

Molecules on the surface of mast cells were measured by flow cytometry. Bone marrow cells that were cultured for 4 weeks were washed with the buffer that was based on phosphate buffered saline (PBS) and contains 0.5% FBS, and scraped with a cell scraper into 1.5 mL tubes to obtain single cells for flow cytometry. 100 μL of cell suspension was incubated briefly for 15 min with 5 μL of Fc blocker (Innovex, USA) at room temperature (RT). Then, 1 μL of monoclonal antibodies PE‐CD117and FITC‐FcεR1α were added and incubated in the dark at 4°C for 30 min at least for analysis after cells were washed once. All of the samples were analyzed using a FACS Calibur cytometer (BD, USA) after cells were washed once. A total of 10,000 events were collected for each sample and data were analyzed with FlowJo software (version 10.0.7r2) with CD117^+^ FcεR1α^+^ representing BMMCs.

### 4.4 Toluidine Blue Staining

The BMMCs were collected after trypsinization, the plates were crawled at 5 × 10^5^ cells/well and spread overnight. Cells were fixed in 95% ethanol for 15 s after washed third in PBS. Then an equal amount of double‐distilled water was added dropwise on the cell climbing slice to mix evenly and stained for 15 min after toluidine blue staining solution (for cells) merely was added dropwise for 5 min. After were washed 3 times with double‐distilled water, the cell climbing slice were removed and air‐dried. Place the cell climbing slice upside down on the slide that was put a drop of neutral resin, and fixed the position around the cell climbing slice with nail polish. The above experimental steps are completed by referring to the description of the toluidine blue staining solution (for cells) reagent. The results are presented through oil lens photography.

### 4.5 ELISA

The BMMCs were stimulated with RAa (3 μM) (He et al. 2017; Shalaby et al. 2017), anti‐DNP‐IgE (300 ng/mL) (Guijarro‐Muñoz et al. 2014; Camp et al. 2015), DNP‐BSA (300 ng/mL), and all trans‐RAL (0.3 μM) (Tang et al. 2018; Torrealba et al. 2020). The BMMCs were divided randomly into different four groups with three sample per group: Control, IgE, IgE/RAL (IR), and IgE/RAL/RAa (IRR). The experimental groups were stimulated selectively with RAa for 0.5 h, then IgE and RAL for 0.5 h, and then DNP‐BSA for 3 h according to different groups (Guijarro‐Muñoz et al. 2014). All reagents were brought to RT and the supernatant was vortexed and centrifuged to make it well mixed before the test. The levels of histamine released from BMMCs were detected by using the Histamine ELISA Detection Kits as per the manufacturer's instructions. The absorbance of each sample was detected on microtiter plate reader. Their concentrations were estimated based on each standard curve, respectively.

### 4.6 qRT‐PCR

The BMMCs were stimulated such as ELISA part said. Trizol reagent was added to the cells to extract total RNA and further reverse transcribed into cDNA. Quantitative real‐time polymerase chain reaction was performed using an Agilent Mx3000P machine according to the reagent instructions. The amount of gene expression levels relative to GAPDH was calculated using the 2^−ΔΔCT^ method in triplicate for each sample. Primers were purchased from Shenzhen Huada Gene Technology Co. (Shenzhen, China) and sequences were as shown in Table 1.

**TABLE 1:** Sequence information of gene primers.

Gene	Primer
*S6K1*	Forward: 5′‐ACA GCC CCG ATG ACT CAA CTC TC‐3′
	Reverse: 5′‐CGT GGG CTA CCA ATA AAT CTT CG‐3′
*IL‐1β*	Forward: 5′‐GCT GCT TCC AAA CCT TTG AC‐3′
	Reverse: 5′‐AGC TTC TCC ACA GCC ACA AT‐3′
*MyD88*	Forward: 5′‐GTT GTG TGT GTC CGA CCG T‐3′
	Reverse: 5′‐GTC AGA AAC AAC CAC CAC CAT GC‐3′
*NF‐κB*	Forward: 5′‐CCA AAG AAG GAC ACG ACA GAA TC‐3′
	Reverse: 5′‐GGC AGG CTA TTG CTC ATC ACA‐3′
*m‐TOR*	Forward: 5′‐GGC TTC TGA AGA TGC TGT CC‐3′
	Reverse: 5′‐GAG TTC GAA GGG CAA GAG TG‐3′
*PI3K*	Forward: 5′‐GCC CAG GCT TAC TAC AGA C‐3′
	Reverse: 5′‐AAG TAG GGA GGC ATC TCG‐3′
*AKT*	Forward: 5′‐AGT CCC CAC TCA ACA ACT TCT‐3′
	Reverse: 5′‐GAA GGT GCG CTC AAT GAC TG‐3′
*TNF‐α*	Forward: 5′‐AGT GAC AAG CCT GTA GCC C‐3′
	Reverse: 5′‐AGC CTT GTC CCT TGA AGA GAA CCT G‐3′
*IL‐4*	Forward: 5′‐TTG CCT TCT TGG GAC TGA T‐3′
	Reverse: 5′‐ATT TCC ACG ATT TCC CAG A‐3′
*IL‐6*	Forward: 5′‐TTG GGA CTG ATG CTG GTG A‐3′
	Reverse: 5′‐GAC TCT GGC TTT GTC TTT C‐3′
*IL‐13*	Forward: 5′‐CTC TTG CTT GCC TTG GTG GTC‐3′
	Reverse: 5′‐AGG GGA GTC TGG TCT TGT GTG AT‐3′
*GM‐CSF*	Forward: 5′‐CAC AAG TTA CCA CCT ATG CGG A‐3′
	Reverse: 5′‐GAG TTC CTG GCT CAT TAC GCA‐3′
*GAPDH*	Forward: 5′‐GAC TTC AAC AGC AAC TCC CAC TC‐3′
	Reverse: 5′‐TAG CCG TAT TCA TTG TCA TAC CAG‐3′

### 4.7 High‐Throughput Transcriptome Sequencing

The BMMCs were stimulated such as ELISA part said. Cells were added to 1 mL of Trizol reagent after cells were washed three times with PBS, then cells were scraped with a cell scraper into 1.5 mL EP tubes to obtain samples. Then samples were handed over to Sangon Biotech Co. Ltd. (Shanghai, China) for high‐throughput transcriptome gene sequencing. Differential expression analysis of genes was performed using OmicShare (version 6.4.5), including heat map, volcano plot, kyoto encyclopedia of genes and genomes (KEGG) pathway enrichment analysis, enrichment circle plot, and gene set enrichment analysis (GSEA) diagrams.

### 4.8 Protein Isolation and Western Blot Analysis

The BMMCs were stimulated such as ELISA part said. BMMCs were harvested and incubated with ice‐cold RIPA lysis (Beyotime, China) and protease inhibitor cocktail tablets (Roche, the USA). After electrophoresis and wet electrotransfer to a PVDF membrane, anti‐phospho(p)‐m‐TOR (1:1000, CST, USA), anti‐p‐AKT (1:1000, CST, USA), anti‐p‐PI3K (1:1000, CST, USA), anti‐p‐p65 (1:1000, CST, USA), anti‐ myeloid differentiation primary response protein 88 (MyD88) (1:1000, CST, USA), anti‐IKKβ (1:1000, CST, USA), anti‐IKBα (1:1000, CST, USA), anti‐p‐ERK (1:1000, CST, USA), and anti‐GAPDH (1:1000, Proteintech, China) were incubated with PVDF membrane at 4°C overnight. Chemiluminescent substrate (ECL, GE Healthcare, the USA) was used for protein visualization.

### 4.9 Statistical Analysis

The above experiments were repeated three times at least. GraphPad Prism software (version 8.0.1) and IBM SPSS 24.0 were used for statistical analysis of the data. All the data were expressed in bar graphs as mean ± standard deviation (SD). Significant differences between the groups statistically analyzed using analysis of variance (ANOVA) and LSD methods, with *p* < 0.05, was considered to indicate a statistically significant difference.

## Author Contributions

**Wenxin Zhang:** formal analysis (lead), investigation (lead), methodology (equal), writing – original draft (lead). **Wenwen Dai:** formal analysis (equal), investigation (equal). **Yingdong Xie:** conceptualization (supporting), writing – review and editing (supporting). **Xingyang Chen:** conceptualization (supporting), writing – review and editing (supporting). **Peng Zhang:** conceptualization (equal), writing – review and editing (equal). **Weiwei Cui:** conceptualization (lead), funding acquisition (equal), writing – review and editing (lead).

## Conflicts of Interest

The authors declare no conflicts of interest.

## Supporting information


**Figure S1.** GSEA diagrams shown the effects of vitamin A deficiency on various metabolism.
**Figure S2.** GSEA diagrams shown the effect of vitamin A deficiency on other functions.

**Table S1.** Differentially expressed genes (DEGs).

## Data Availability

The data that support the findings of this study are available from the corresponding author upon reasonable request.
