# Supplementary material for: Retinoic Acid Regulates Allergic Inflammation via Limiting Mast Cell Activation
Source: Food Sci Nutr. 2025 Jan 7;13(1):e4727. doi: 10.1002/fsn3.4727 (PMC11717043; doi:10.1002/fsn3.4727)
Supplement: Supplementary file 1 — Figure S1. GSEA diagrams shown the effects of vitamin A deficiency on various metabolism. Figure S2. GSEA diagrams shown the effect of vitamin A deficiency on other functions. [file FSN3-13-e4727-s001.docx]

**Figure S1**


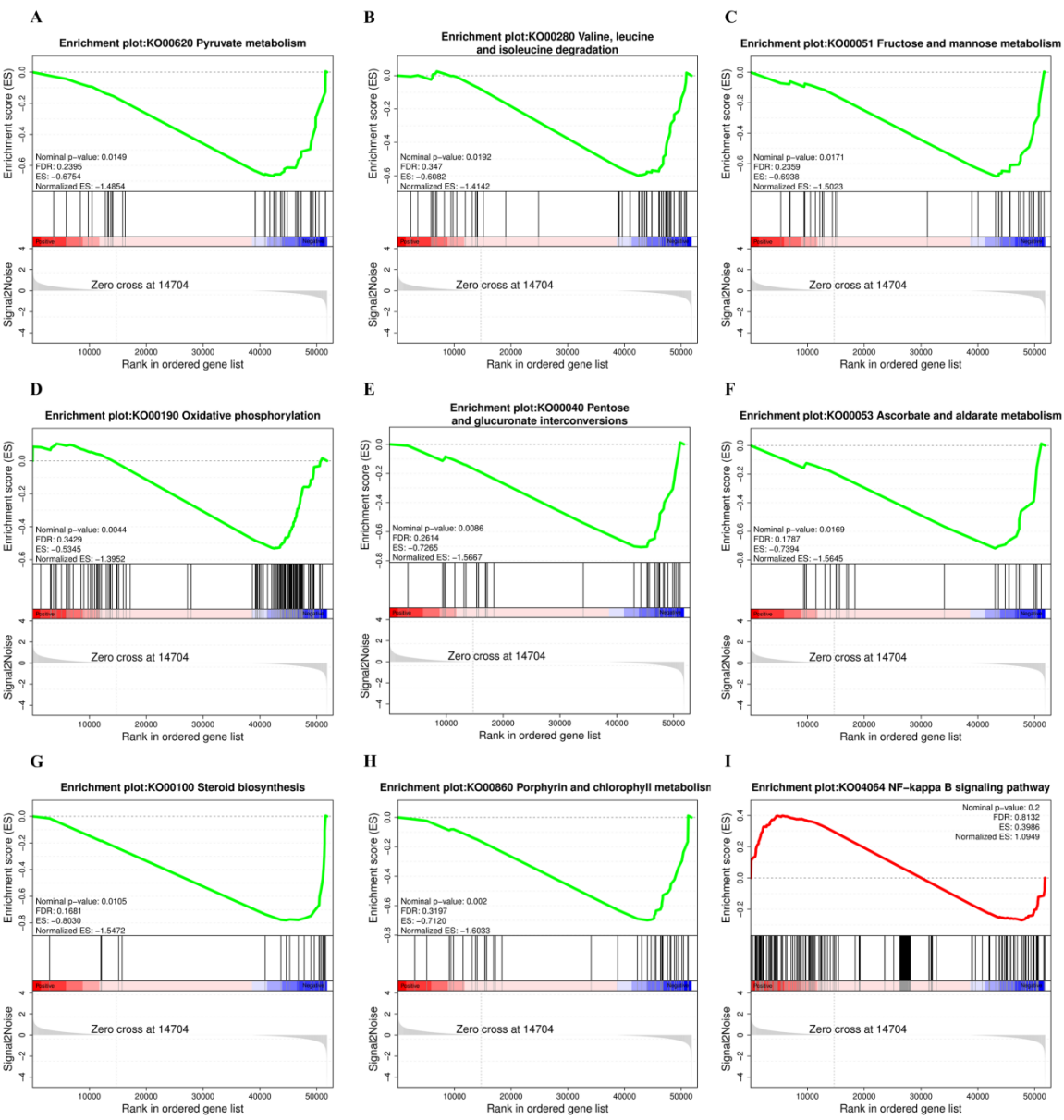


**Figure S1 GSEA diagrams shown the effects of vitamin A deficiency on various metabolism.**

(A-H) GSEA diagrams of changes in various metabolic aspects in the two groups (i.e. IR group and IRR group). The experiments were repeated three times independently, and the up-regulated genes and down-regulated genes were highlighted in red and blue. (I) A GSEA plot of differences in gene expression in the NF-κB signaling pathway between the two groups.

**Figure S2**


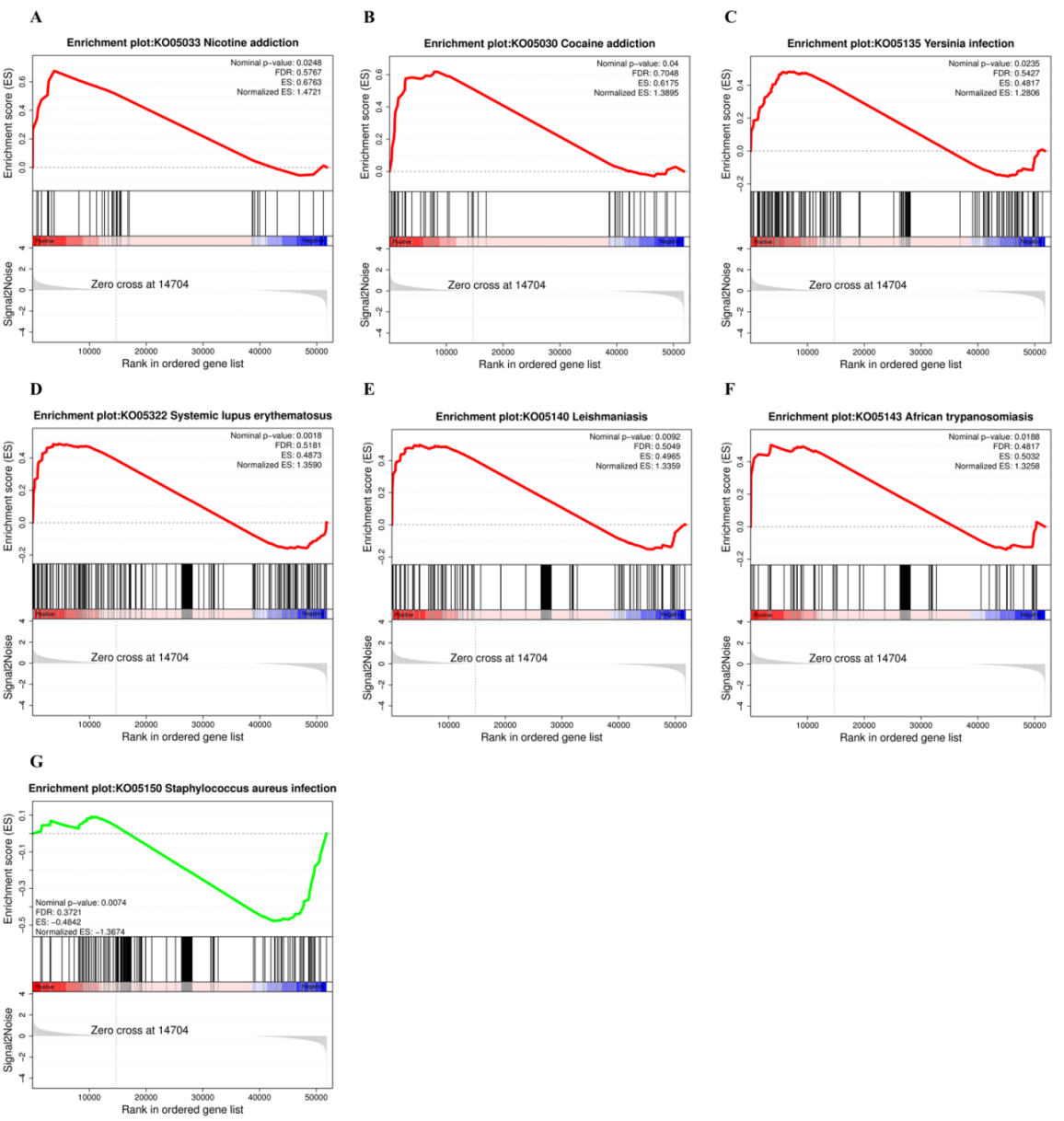


**Figure S2 GSEA diagrams shown the effect of vitamin A deficiency on other functions.** (A-G) GSEA diagrams of the effects in two groups (IR group and IRR group) on other functions. The experiments were repeated three times independently, and the up-regulated genes and down-regulated genes were highlighted in red and blue.
